# Surface Protection of Quaternary Gold Alloys by Thiol Self-Assembled Monolayers

**DOI:** 10.3390/ijms232214132

**Published:** 2022-11-16

**Authors:** Guadalupe Sánchez-Obrero, Irene Humanes, Rafael Madueño, José Manuel Sevilla, Teresa Pineda, Manuel Blázquez

**Affiliations:** Department of Physical Chemistry and Applied Thermodynamics, Institute of Chemistry for Energy and Environment, University of Cordoba, Campus Rabanales, Ed. Marie Curie, E-14014 Córdoba, Spain

**Keywords:** quaternary gold alloys, self-assembled monolayers, cyclic voltammetry, electrochemical impedance spectroscopy, surface characterization, jewelry, biomaterials applications

## Abstract

This work deals with a physical and chemical surface characterization of quaternary 18K, 14K, and 9K gold alloys and pure polycrystalline gold substrates. Surface microstructure and composition are evaluated by scanning electron microscopy (SEM), X-ray photoelectron spectroscopy (XPS), and X-ray fluorescence spectroscopy. Corrosion resistance of 18K gold alloys is explored by potentiodynamic polarization showing the influence of the manufacturing process on materials fabricated as plates and wires. The research is also in the framework of one of the most common strategies on the modification of metallic surface properties, i.e., the building of self-assembled monolayers (SAM) from organic thiols. The metal affinity of the head group to produce the coating of the substrate by covalent binding is approached by using thiol compounds with different molecular structures and functional group chemistries exposed to an electrolyte solution. Therefore, a comparative study on the surface protection of a quaternary 18K gold alloy and pure gold substrates by SAMs of 6-mercaptopurine (6MP), 1-decanethiol (DT), and 11-mercaptoundecanoic acid (MUA) has been carried out. Surface modification and SAM organization are followed by cyclic voltammetry (CV), and the behavior of the double layer of the electrode–electrolyte interface is evaluated by electrochemical impedance spectroscopy (EIS). The study of these materials allows us to extract fundamental knowledge for its potential application in improving the bioactive properties of different jewelry pieces based on 18K gold alloys.

## 1. Introduction

Gold is used in medical practice for dental restorations, implants, and prostheses [1], and it is also an especially featured material in the jewelry industry. Gold is characterized by its biocompatibility and superior corrosion resistance [2]. As gold is expensive, its clinical use has been mainly limited to dental implants and, to a minor extent, wire fixings and supports, ocular prosthetics, endovascular stents, soluble injectable compounds to alleviate rheumatoid arthritis, drug delivery via nanoparticles, among others [3,4,5].

The use of noble metals in jewelry is well-known. However, 24-carat (24K) pure gold is not widely used in most jewelry objects, and it is mainly found mixed with non-negligible proportions of other metals like Ag, Cu, Co, and Zn to form quaternary alloys (e.g., 18K, 14K, and 9K), to improve its strength and malleability. The interaction of both pure gold and gold alloys in tissue and skin is also known to occasionally produce allergic-type reactions and other toxic effects [6,7].

Gold has a standard potential of +1.83 and +1.52 V versus the normal hydrogen electrode (NHE) for Au^+^ and Au^3+^ species, respectively, which leads to a stronger resistance corrosion than the rest of the components present in the alloys. However, under extreme experimental conditions (e.g., strong saline solutions that simulates sweat). the formation of gold complexes and gold trichloride becomes thermodynamically stable. This would agree with reports where gold cations may be the reason for allergic and other toxic activities in the body [8]. In this sense, Au alloys in jewelry objects that are in close contact with the skin and exposed to pH and saline environmental conditions typical of body sweat may suffer corrosion, and occasionally produce undesirable allergic reactions.

Materials that are biocompatible, but also long-lasting and wear-resistant, are factors appreciated by jewelry customers. In fact, gold alloys used for jewelry must meet quality standards on hardness; corrosion mechanical stress; absence of allergic reactions; abrasive, frictional, and mechanism wear [9,10,11,12,13,14,15]; adequate color design [16,17,18,19]; and brightness [20]. Thus, corrosion studies based on quaternary alloy degradation and modification of their interfacial surface properties, while keeping the quality standards demanded on jewelry, are of fundamental interest in the context of controlling its bioactivity.

In the last decades, the modification of metal surfaces has been carried out by using self-assembled monolayers (SAMs) endowing substrates with specific and tunable surface properties oriented toward the design of technological and smart functional interfaces [21,22,23,24,25,26,27,28,29,30,31,32,33,34,35,36,37,38]. SAMs, due to the balance of intermolecular interactions, head group–substrate binding, pending tail-groups; its molecular structure and monolayer architecture act as a molecular barrier that can tailor hydrophobicity and hydrophilicity, wettability, conductivity, and electronic properties either of metals [21,22,23,24,32] or alloys [39,40,41,42,43,44,45,46,47,48].

SAMs have been considered as a prototype of nanotechnology [22]. The organic molecules that form the SAM carry on with the “instructions” required to spontaneously generate an ordered and nanostructured material on surfaces. The final structure of the SAMs are the result of a complex molecular organization within quasi-equilibrium 2D assemblies by the interplay of thermodynamic and kinetic factors, which will determine their final properties and function [21,22,23]. SAMs demonstrate that molecular-scale design offers a wide-spread platform and versatile approach for surface functionalization. Among SAMs, thiol-based monolayers built on gold single crystals are archetypical systems to investigate the effect of surface crystallinity and specific structural features of the molecular backbone and/or the end-group on their organization at the molecular level [24].

Then, surface modification should aim not only to improve biocompatibility of metallic materials for biomedical use [1,2,5], but also in other fields of application. In this sense, the use of thiols to form SAMs and protect alloy surfaces offers an opportunity to tailor the bioactivity of jewelry products while maintaining their standards. The present work will explore the surface modification effect on a quaternary gold alloy purchased from a local jewelry company by using three thiol compounds with different molecular structures, based either on aliphatic chains or aromatic ring backbones, and functional terminal moieties, such as carboxy or methyl groups. The candidates chosen for the SAM formation to protect and modify the interfacial properties of the alloy surface included 6-mercaptopurine (6MP), 1-decanethiol (DT), and 11-mercaptoundecanoic acid (MUA). On gold, 6MP monolayers are known to be very sensitive to surface atomic order and grain boundaries [25], and they assemble in a very short time (≈2 min) due to strong stacking intermolecular interactions. Moreover, 6MP and MUA SAMs become ionizable, depending on pH experimental conditions [30]. On the other hand, DT and MUA monolayers produce closed-packed and well-organized structures onto the gold substrate upon longer immersion times ranging from hours to days. This fact provides improved insulating and blocking/barrier properties, together with a wider potential stability range toward direct or mediated electron transfer of chemical or biochemical agents, where model interfacial behavior may be observed depending on chain length and polarity of the terminal groups of the SAMs.

First, we aim to study the physical and chemical surface properties of several quaternary gold alloys used in jewelry (18K, 14K, and 9K). For this purpose, corrosion resistance, material aging, surface composition, and microstructure imaging of the bare substrates were characterized by scanning electron microscopy (SEM), X-ray photoelectron spectroscopy (XPS), X-ray fluorescence spectroscopy, and electrochemical polarization curves. Corrosion curves were obtained in a saline solution that simulated sweat. In the case of the 18K gold alloy, the influence on surface microstructure and corrosion resistance of two different types of substrates were also examined, according to its manufacturing process that produces either wire or plate pieces. Finally, the 18K gold alloy was modified with three different kinds of SAMs to protect the surface without altering the composition and physical properties (e.g., colour, brightness, hardness, etc.) of the materials demanded by the jewelry market. A comparative electrochemical study by cyclic voltammetry (CV) and electrochemical impedance spectroscopy (EIS) was carried out for a bare and SAM-protected 18K gold plate alloy to analyze the effect of the functionalization, in prospect of the surface bioactivity of these materials that may be altered in sweat-simulated solutions. Procedures and methodologies are shown here to endow a gold alloy with specific interfacial properties depending on the SAM formed as a proof-of-concept to help in acquiring knowledge and producing modified materials that may be selected for future studies on bioactivity with possible practical applications in jewelry materials.

## 2. Results and Discussion

### 2.1. Surface Characterization of Gold Alloys

**Figure 1:**
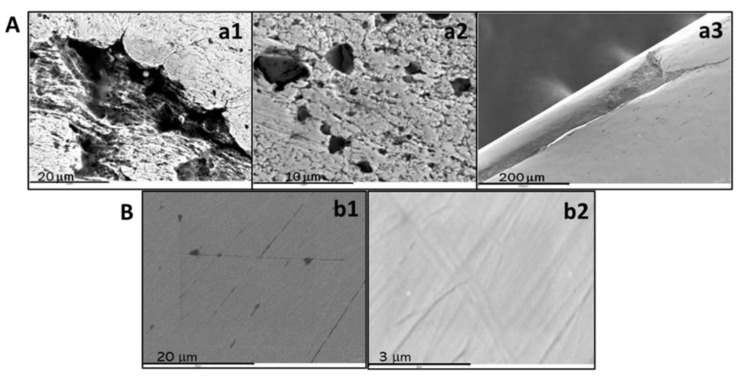
SEM images of a commercial 18K gold quaternary alloy (Au-Ag-Cu-Zn) wire (**A**) and plate (**B**). Hollows caused by shrinkage (a1), cooling (a2), and fracture by stretching (a3). Surface micrographs at low (b1) and high magnification (b2).

Quaternary gold alloys (Au-Ag-Cu-Zn) of different shapes and compositions showed a similar macroscopic appearance. However, different features could be found in SEM micrographs for samples manufactured with distinct geometries. Plate surfaces were very smooth, though they showed thin linear scratches probably produced by the polishing treatment. Wire surfaces had quite a few flaws as grains and microscopic clefts with dendritic structures were probably induced by air bubbling during the melting treatment. In this sense, cold stretching to produce thread thickness may also lead to fractures, as those observed in Figure 1.

Dispersive energy microanalysis based on SEM-EDX [49] allows for the comparison of the sample surface composition of the different alloys (Table 1). The gold compositions was very similar to that obtained by the standard official methods used in jewelry, such as X-ray fluorescence (XRF) and cupellation [50,51]. Therefore, the samples studied here contained at least the minimum gold composition of 75, 58.3, and 37.5%, admitted for 18, 14 and 9 carat Au alloys, respectively.

Likewise, X-ray fluorescence (XRF) was used to validate the composition of quaternary alloys (Table 2), and these results were consistent with those obtained by SEM-EDX.

**Figure 2:**
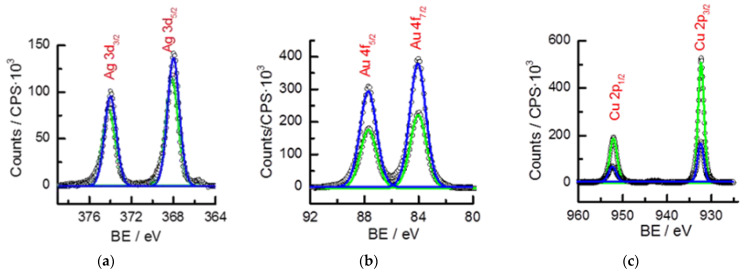
High-resolution XPS of quaternary gold alloys (black circles) of the (**a**) Ag, (**b**) Au, and (**c**) Cu bond energies. Regions of Ag3d, Au4f, Cu2p, and spin-orbit coupling. Deconvolution was obtained by Gaussian/Lorentzian curves. Gold alloy Au 18K (blue) and Au 9K (green).

XPS surface analysis confirmed the presence of Au, Ag, Cu, and Zn as the main elements, such as in 18K and 9K alloy samples [49]. In this case, Cu, Ag, and Au XPS signals clearly showed changes on the proportion of some elements (Figure 2). High-resolution spectra make it possible to estimate the percentage of sample components. However, none of these methods were officially adopted to accurately validate the overall composition, as SEM-EDX and XPS measurements were restricted to a surface layer of several micrometres and nanometres thick, respectively. Therefore, although such methods may be used for the characterization of the material composition, the reference method accepted in jewelry is the fire assay [51]. The percentage of Zn determined by XPS differed from EDX and XRF measurements, most likely due to its low content and a probable alteration in the surface enrichment of other components at low-content gold alloys (Table 3) [52].

### 2.2. Polarization Curves

**Figure 3:**
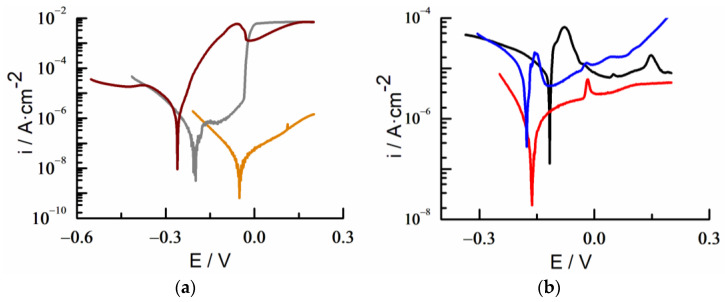
Polarization curves for wire surfaces of (**a**) pure copper (wine), silver (grey), and gold (orange), and (**b**) 18K (black), 14K (red), and 9K (blue) gold alloys in NaCl 3.5%.

Polarization curves were recorded in a standard saline solution of 3.5% NaCl to observe durability and resistance of the gold alloys. The study includes samples of its major components, Au, Ag, and Cu, as pure metals. The samples were kept in solution at open circuit (OC) during the time required to reach an equilibrium potential (OCP). Potentiodynamic curves were obtained in a potential range centered at the OCP value. The scan rate was 1 mV/s and curves were plotted as log (j/μA·cm^−2^) vs. E (mV), allowing for the determination of Tafel slopes, corrosion potential (E_corr_), and current (I_corr_) of the samples [53,54,55,56] (Figure 3).

The corrosion rate of quaternary gold alloys increases by up to an order of magnitude compared with that of the pure metals. This increase in I_corr_ is not due to the composition of the alloy, but rather, it seems to be related to the surface boundaries of the different metal domains present in the surface mixture of the gold quaternary alloys [57]. Tafel slopes did not follow any pattern related to the composition or any other tangible characteristic of the alloys (Table 4), suggesting a more complex behavior that needs to be further analyzed in more detail.

The values of E_corr_ in the alloys were lower than those corresponding to pure gold, but higher than the pure Ag and Cu ones, increasing their value when metal nobility also increased according to their thermodynamic stability (Table 4) [58].

**Table 4:** Parameter for metals and gold alloys in 3.5% NaCl.

Alloy or Pure Metal	Ecorr/mV	Icorr/µA·cm^−2^	Anodic Tafel/mV	Ecorr/mV
Au	50	0.029	87	0.160
18K	−115	7.267	166	0.015
14K	−160	1.864	130	0.997
9K	−180	4.451	56	0.037
Ag	−200	0.055	77	0.051
Cu	−260	0.834	32	0.028
Zn ^1^	−1005	0.5–1.0		

^1^ [59].

**Figure 4:**
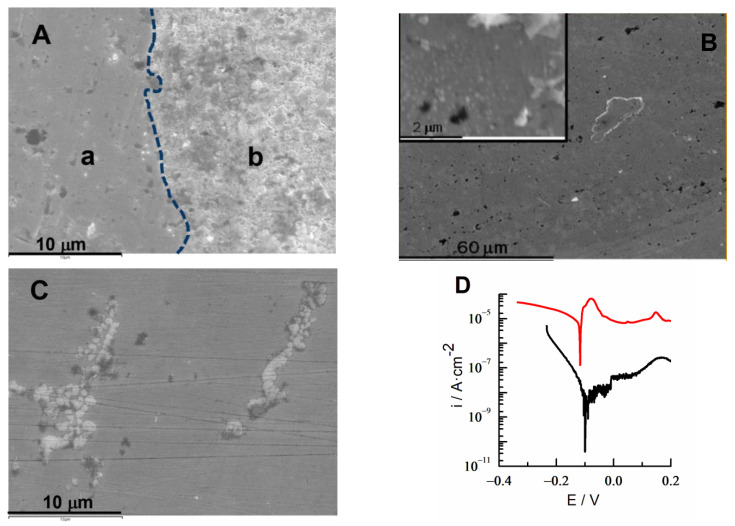
SEM micrographics of an Au 18K gold alloy after anodic polarization in 3.5% NaCl. (**A**) High polarization potential where the unexposed (**a**) and exposed (**b**) corrosion wire areas are shown. (**B**) Intermediate polarization potential with barely appreciable corrosion. Inset: zoom in image. (**C**) Effect of three successive polarization scans up to potential of a plate. (**D**) Comparative polarization curves of wire (red) and plate (black).

After polarizing a 18K gold alloy to anodic potentials, the substrate surface was inspected by SEM. Figure 4A shows an image of the boundary region that delimits both non-immersed and immersed zones of the substrate in a saline solution. A significant increase in roughness was observed at both low- and high-resolution images for high anodic polarization potentials (E > E_corr_, i.e., E close to +1 V). SEM-EDX analysis in the corrosion zone showed the presence of chloride ions and a decrease in the Au surface composition concomitantly with the precipitation of salt microcrystals on the surface (Figure 4A,B).

However, when polarization was applied within a lower potential range, i.e., to intermediate anodic potentials, E > E_corr_, about E~+0.2 V, there was barely any surface effect found, as described above (Figure 4B inset). The corrosion seems to occur in localized areas, being the aspect of the substrate, at low resolution, that is very similar to that of an unpolarized region.

When this study was carried out under the same experimental conditions using a plate instead of a wire, no sign of corrosion appeared until the polarization scan was repeated several times at intermediate potentials, E > E_corr_, E~+0.4 V (Figure 4C). This indicates that the corrosion pitting of the surface not only depends on alloy composition, but also on the manufacturing process. These observations were corroborated by SEM-EDX images and by polarization curves. I_corr_ differs by more than two orders of magnitude between both types of surfaces, ranging from 7.267 (Figure 3, Table 4) to 0.046 µA/cm^2^ for the wire and the plate objects, respectively (Figure 4D—average values).

### 2.3. Electrochemical Characterization of the Quaternary 18-Carat Gold Alloy

**Figure 5:**
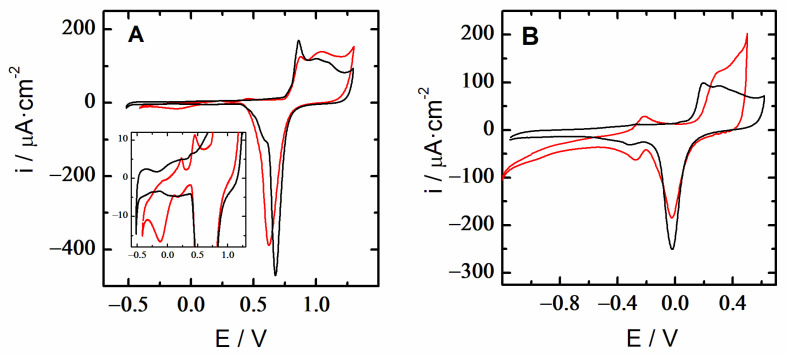
Cyclic voltammetry of 18K quaternary gold alloy (red) and pure polycrystalline gold (black) in (**A**) 10 mM HClO_4_ and (**B**) 0.1 M NaOH. Scan rate 100 mV/s.

A comparison of the electrochemical i-E profiles of the 18-carat quaternary gold alloy and polycrystalline gold electrodes was carried out in 10 mM HClO_4_ and 0.1 M NaOH aqueous solutions (Figure 5). The polycrystalline gold response in an acid medium showed only gold surface oxidation during the anodic scan and stripping of the gold oxides in the reverse one. The i-E response of the 18K gold electrode followed a similar pattern, but it was additionally observed for a faradaic current in the double layer region. A closer look at this region showed the presence of a pair of voltametric peaks centered at 0.35 and 0.1 V, which could be assigned to silver and copper, respectively [60]. In a basic medium, such differences between both gold and alloy substrates were emphasized. Thus, the electrochemical response was sensitive to the alloy composition, consistent with the fact that only 75% of the content of the tested samples was gold.

**Figure 6:**
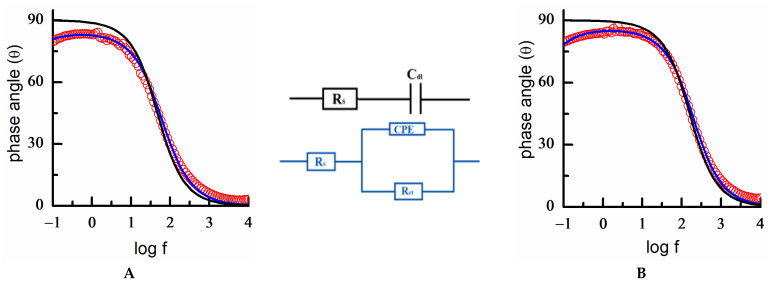
Electrochemical impedance spectra. Experimental Bode plot data of a square plate of 18K gold alloy (red circles) in (**A**) 10 mM HClO_4_ and (**B**) 0.1 NaOH. Experimental data (red circles) are fitted to a [R(QR)] equivalent circuit (blue lines). A simulated RC equivalent circuit is included for comparison (black lines). E = −0.3 V. Schemes of the RC and [R(QR)] circuits are depicted.

A study of the interfacial properties was performed by electrochemical impedance spectroscopy (EIS), both in acidic and basic media. The EIS analysis of 18K gold alloys revealed that the double-layer region did not behave as an ideal capacitor [61]. In this case, a typical experimental Bode plot showed a clear deviation at low frequencies from 90°, the theoretical value of the phase angle for a capacitor (Figure 6). Therefore, the experimental data did not fit to a pure series RC equivalent circuit, and it was necessary to replace the ideal capacitor by a constant phase element (CPE or Q [62]). Additionally, as in the present system, adding a parallel resistance to properly simulate the interphase was also required, which was represented by a R(QR) equivalent circuit, where the parenthesis indicates a parallel disposition of both R and Q elements in series with the solution resistance. This feature shows a non-ideal behavior of the interphase, and clearly indicates the lack of homogeneity of the alloy surface compared with the gold polyfaceted surface [61,62].

### 2.4. Surface Functionalization of the 18-Carat Gold Alloy

**Scheme 1:**
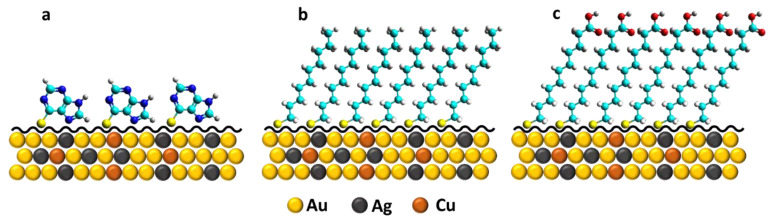
SAM interfaces built-in on 18K gold alloy surfaces: (**a**) 6MP, (**b**) DT, and (**c**) MUA. Zn atoms are not shown considering its surface proportion is below the minimum threshold of the total atoms depicted here.

The 18-carat gold alloy was modified by anchoring molecular self-assembled monolayers on its surface. Apparently, functionalization by chemical modification of the alloy surface with 6-mercaptopurine (6MP), decanethiol (DT), and mercaptoundecanoic acid (MUA) SAMs does not imply any changes in brightness, colour, and external aspects of the substrate. The main motivation for the construction of these electrochemical interfaces is the opportunity to obtain surfaces with different blocking and physicochemical properties, as well as exploring electrode–electrolyte interactions that may be tuned by ionizable terminal groups (Scheme 1). The molecule 6MP is an aromatic thiol that attaches to the metal surface, interacting with sulphur and a pyrrolic nitrogen atom by exposing the purine ring with conductive, polar, and pH-sensitive properties [25,26,28,30]. DT and MUA are ω-substituted alkanethiols that allow adsorption and assembly on the surface, exposing respective -CH_3_ and -COOH groups toward the solution [63,64]. Further investigation of SAM under several experimental conditions should be conducted for a more precise diagnosis of the modification effect they can produce on barely explored heterogeneous metallic surfaces, such as alloys [65,66,67,68].

**Scheme 2:**
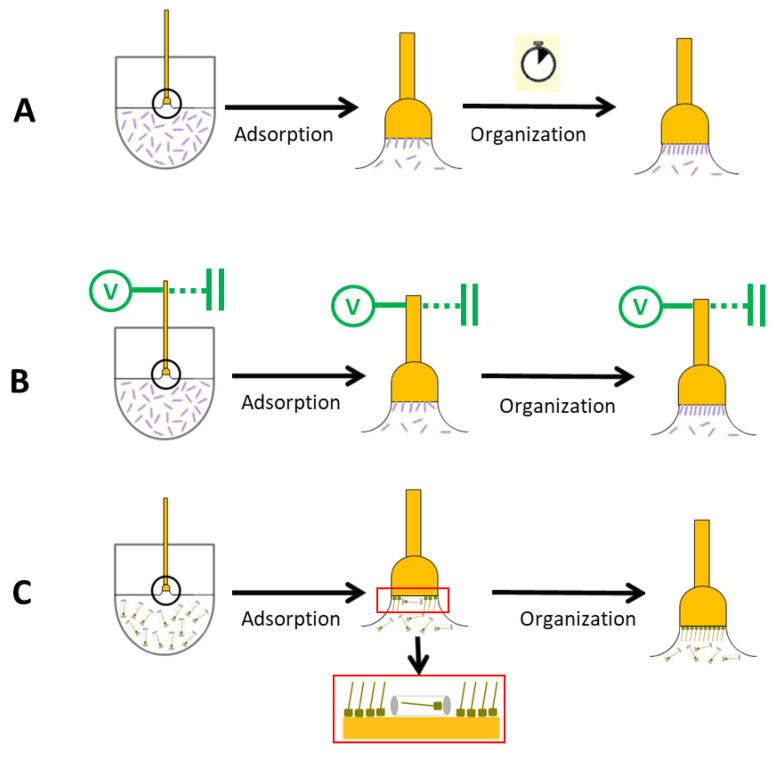
SAM formation approaches by dipping in ethanolic or aqueous solution (**A**), under electrochemical control (**B**), and in a micellar medium (**C**).

The formation of monolayers followed some of the procedures depicted in Scheme 2, where SAMs were prepared by dipping the substrate into: (a) an ethanolic or aqueous solution containing the mercaptoderivative compound [21,25,26,27,28,29,30], (b) an aqueous solution under electrochemical control [31,63], and (c) a micellar lyotropic medium that encapsulates and delivers the modifier molecules to yield highly-packed SAMs with better blocking properties at a lower processing time [31,64,69].

### 2.5. Mercaptopurine SAM

The 6MP-SAM was formed by immersion of an 18-carat gold alloy sample in an aqueous diluted acidic solution (100 μM 6MP, HClO_4_ 0.01 M) during a time interval that varied from two minutes to several hours. An optimum immersion time of 10 min was selected to completely assure the formation of a highly ordered 6MP monolayer. One of the most common ways to characterize SAMs is the use of electrochemical methods. A scan to negative potentials from the double-layer region allowed for observation of the reductive desorption of the 6MP-SAM modified gold electrode, a special case of SAM that is extremely sensitive to surface atomic order and crystallinity [25,28]. The modified gold electrode was immersed in an alkaline solution, free of adsorbate, which usually led to multiple peak-shaped signals in cyclic voltammetry, due to surface-confined reduction reactions ascribed to the S-Au bond breakage taking place at different crystallographic surface sites. Reversing the potential may produce the re-adsorption of the thiols on the metal surface that were not able to diffuse far away enough from the interfacial layer. The reductive desorption process (RD) of the molecules bound by a metal–sulphur bond allows for an estimation of the thiol surface coverage and SAM packing, according to Equation (1).

R − S − M + *solvent* + 1e^−^ → M*solv* + R − S^−^*solv* (1)

where M*solv* and R–S^−^*solv* stand for the solvated metal surface and thiolate molecules, respectively. This equation represents a solvent substitution reaction that considers the energetic contributions involved in SAMs RD, such as substrate–adsorbate, adsorbate–solvent, neighboring adsorbate–adsorbate, substrate–solvent, and surfactant solvation interactions [70]. In this sense, the charge density contains not only the faradaic charge due to the RD, but also the double-layer charging contribution. Moreover, the stoichiometry of Reaction (1) to account for the RD process and the charge flowing through the interface per desorbed molecule is not an integer equal to the number of electrons transferred from metal to the molecule, but depends on electrode potential and the nature of the supporting electrolyte [71].

Studies of the mechanism suggest that desorption starts on defects and grain boundaries, and it continues growing at nucleation sites that are randomly distributed across organized regions of the SAM [27,28,29]. The desorption rate induced by the electric field seems to be higher for the adsorbate molecules located at SAM domain boundaries or defects. The desorption potential depends on factors such as the hydrocarbon chain length and its degree of structural order due to intermolecular interactions stabilizing the organic monolayer, as well as the crystallinity of the substrate [21,22,23,24].

**Figure 7:**
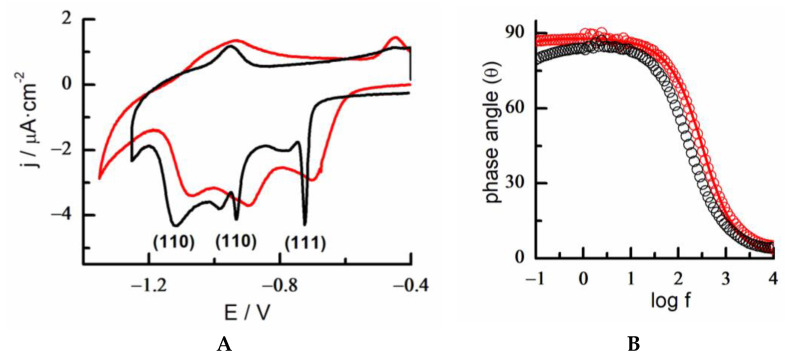
(**A**) Cyclic voltammograms of the reductive desorption of 6MP-SAM in KOH 0.1 M on Au 18K gold alloy plate (red) and polyfaceted gold bead (black). Scan rate 0.02 V/s. (**B**) Electrochemical impedance spectra. Bode plot of a 1 × 1 cm^2^ square plate of 18K gold alloy. Variation of the phase angle with the frequency of the bare alloy (black circle) and 6MP-SAM/alloy (red circle). E_dc_ = −0.3 V. Fitting of experimental data to a [R(RQ)] equivalent circuit (red line).

Figure 7A shows the RD of a monolayer of 6MP in 0.1 M KOH, which was assembled after a modification time of 10 min. As commented above, the removal of the 6MP monolayer from the surface causing breakage of the S-Au bond allowed us to obtain information of the coverage, degree of molecular organization, and surface crystallinity of the substrate [25,28].

Oxidative adsorption and reductive desorption of the 6MP are driven by structure-sensitive processes. Therefore, the i-E profile of the reductive desorption of the 6MP-SAM on poly-oriented gold presented several desorption peaks that were very narrow at well-defined potentials. These features could be explained considering S-Au bond breaking from different crystalline orientations, including the low index Au(111), Au(100), and Au(110) facets (Figure 7A) [25]. In 18K gold plate alloys, broader peaks were clearly observed with a displacement of approximately 50 mV to lower energies. This behavior was also corroborated at lower-content gold alloys (data not shown). The presence of narrow CV spikes was indicative of strong lateral interactions between surface-assembled 6MP molecules, due to the formation of long-range highly ordered structures onto atomically flat terraces with different crystallographic orientations. Therefore, the RD of the monolayer from the 18K gold alloy was consistent with a lower degree of long-range structural organization of the 6MP-SAM by the presence of silver and copper on the surface composition. This fact may be a consequence of higher Au segregation into smaller size domains and/or different atom bonding distances (e.g., as isolated atoms or cluster areas coordinated or surrounded by the rest of the components) at the alloy surface that disrupted effective stacking ring interactions between neighboring molecules.

Despite this effect, the charge density obtained in the desorption process on 6MP-Au 18K was about 60 μC/cm^2^, similar to that obtained for a complete monolayer of 6MP on polycrystalline and single crystal gold substrates (surface coverage, θ = 0.25, n = 1 e^−^) [25,28], i.e., it could be deduced that a fully covered and well-protected surface with 6MP-SAMs was obtained on this heterogeneous alloy surface.

A non-destructive complementary EIS assay of the 6MP monolayer allowed us to determine the effect of the modification on interfacial properties and surface integrity. Selecting a potential in the double-layer stability region of the SAM on the modified Au 18K plate, the EIS response showed a different behavior compared with that of the unmodified surface (Figure 7B). The EIS spectrum of the 6MP modified alloy was now well-fitted to an ideal capacitor model (RC equivalent circuit), with a double-layer capacitance value determined to be 17 μF/cm^2^, which is in good agreement with that found for a 6MP-SAM [28,30]. In this way, the successful functionalization of the alloy surface with a molecular monolayer of an approximate thickness comprised between 6–8 Å largely eliminates the interfacial effect of surface heterogeneity. In summary, it can be noted that 6MP plays a dual role in binding to the Au-18K surface. On one hand it forms a complete protecting monolayer and, on the other hand, it produces a homogenizing effect on the modified surface.

### 2.6. Decanethiol SAM

**Figure 8:**
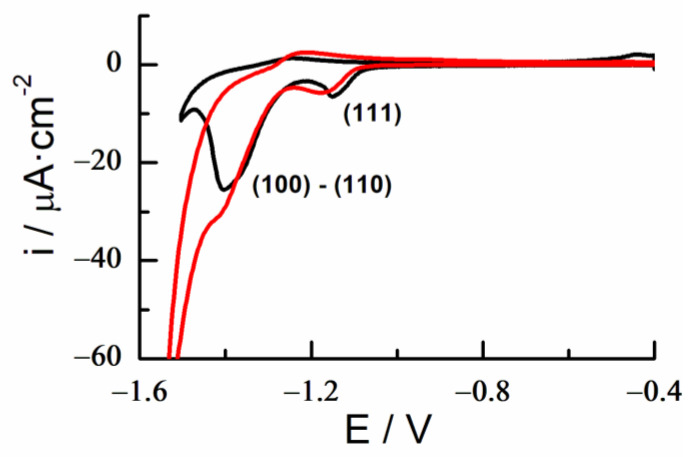
Reductive desorption. Cyclic voltammetry of decanethiol SAM in KOH 0.1 M of a gold 18K plate (red) and pure gold (black). Scan rate 0.02 V/s.

A decanethiol SAM (DT-SAM) was deposited by dipping a clean electrode in an ethanolic solution of DT 1 mM, which was assembled after a modification time of 2 h. Figure 8 shows the voltammetric profile of the dissolution of a DT-SAM on Au-18K in an alkaline solution compared with that of a pure gold substrate. The charge density for n-alkanethiol and α,ω-alkanethiol SAMs ranged from 75–85 μF/cm^2^ (surface coverage, θ = 0.33, n = 1 e^−^) on gold for a (√3 × √3) R30° structure of closely packed ordering of molecules, with the non-polar hydrocarbon chain fully extended in an upright configuration [21,22]. The charge density obtained for a DT-SAM/Au18K was approximately 90 μC/cm^2^, corresponding to a complete surface coating and a similar DT surface organization as obtained on pure gold. This is a clear indication that the alloy surface can be efficiently functionalized.

**Figure 9:**
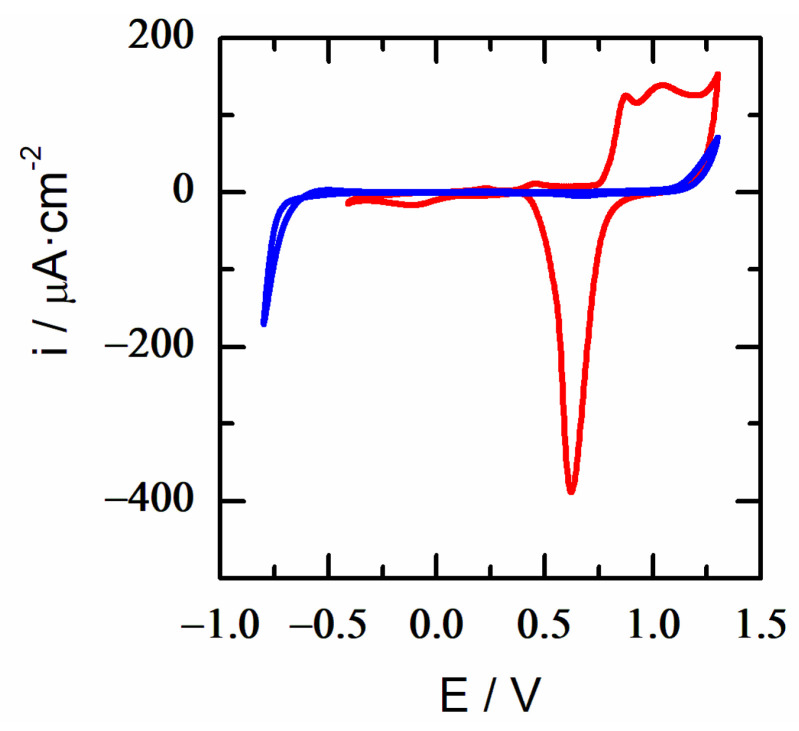
Surface protection. Cyclic voltammetry of Au 18K (red) and DT-SAM Au 18K (blue) in 0.01 M HClO_4_. Scan rate 0.02 V/s.

As it happens with 6MP monolayers on Au 18K, it is not possible to reductively desorb the DT layer from this surface in an acidic medium without concomitantly causing proton reduction from the aqueous medium at sufficiently negative potentials (Figure 9). However, the presence of the DT monolayer can be easily detected under these conditions because the DT-SAM protects the substrate in a wide range of potentials, preventing surface faradic reactions within the stability zone of the monolayer. Such anodic/cathodic stability potential regions range from +1.0 to −0.5 V. Beyond these limits, the onset of the oxidative and reductive desorption of the monolayer appears, together with the surface oxidation of the alloy and/or the hydrogen evolution reaction at higher overpotentials (~up to 0.5 V), respectively.

**Figure 10:**
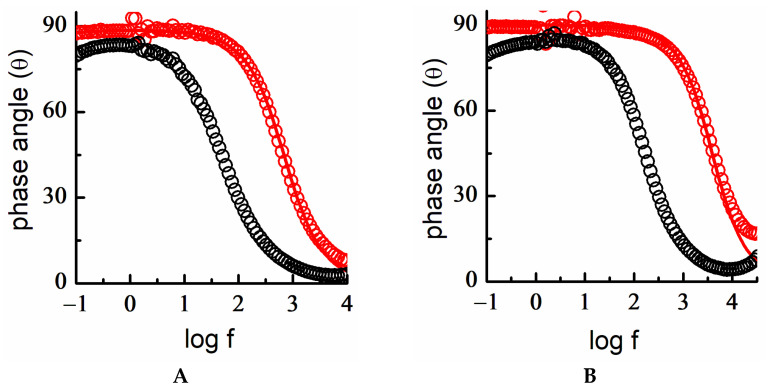
Electrochemical impedance spectra. Bode plot of bare Au 18K gold alloy (black circle) and DT-SAM/Au 18K (red circle) in (**A**) acidic medium, E = 0 V, fitting of experimental data to a [R(RQ)] equivalent circuit (red line) and (**B**) basic medium, E = −0.3 V, fitting of experimental data to a [RC] equivalent circuit (red line).

The EIS study of DT-SAMs on Au 18K in the potential region of the double-layer again shows almost ideal behavior, both in acidic and basic media, with phase angle values very close to 90° in a wide range of frequencies (Figure 10). Therefore, these findings corroborated that surface functionalization eliminated the effect of surface heterogeneity on bare quaternary alloys of an 18K gold plate. The low capacitance values determined for the modified interface, C_dl_ (SAM) < 2 μF/cm^2^, corresponded to those of a highly packed organized hydrophobic DT monolayer in a fully extended configuration of alkyl chains and a thickness of approximately 12 Å, which exhibited good insulating properties [31,64,69]. Thus, the DT-SAM shows excellent blocking and barrier behavior against reactions occurring at this alloy surface under both acidic and alkaline conditions.

### 2.7. Mercaptoundecanoic Acid SAM

The formation of MUA-SAMs was carried out from a lyotropic phase solution. The deposition solution was formed by mixing Triton X-100 (42%) and water (58%). The mixture was heated until the lyotropic phase (40–45 °C) was obtained, and then MUA was added while stirring to obtain a homogeneous mixture of a 1 mM final concentration. Then, the temperature was cooled down to 28 °C and kept constant during the SAM formation. After the immersion time, the micellar solution was removed, and the modified substrate was thoroughly cleaned with distilled water [64,69].

**Figure 11:**
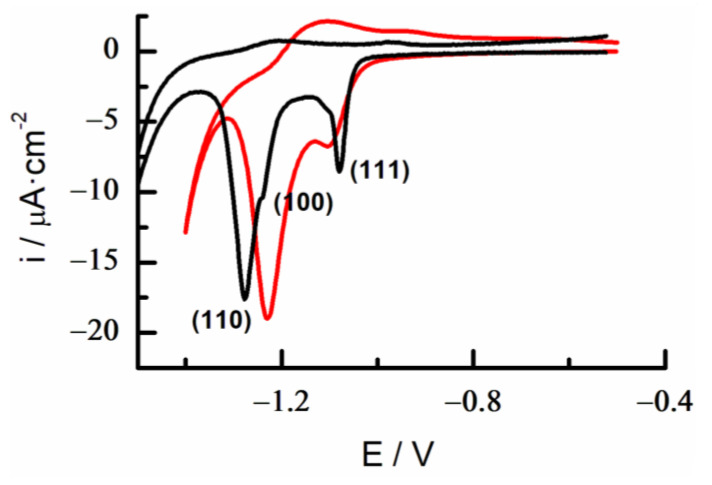
Cyclic voltammograms of the reductive desorption of MUA-SAMs on Au 18K (red) and polyfaceted gold (black) in KOH 0.1M. Scan rate 0.02 V/s.

The reductive desorption of the MUA monolayer prepared upon a modification time of 10 min was characterized in 0.1 M KOH (Figure 11). The i-E profile in cyclic voltammetry showed two peaks corresponding to the MUA desorption at low-index facets (Figure 11). The definition and separation of these peaks was somewhat lower in the (111) facet of the modified alloy than in the polyfaceted gold electrode. It was also observed that the small shoulder assigned to (100) facets disappeared in the case of the modified gold alloy. As already described for 6MP-SAMs, such behavior suggests that long-range order interactions at Au low index terraces are limited in this quaternary alloy compared with the polyfaceted gold substrate, due to the presence of surface atoms of the other components.

**Figure 12:**
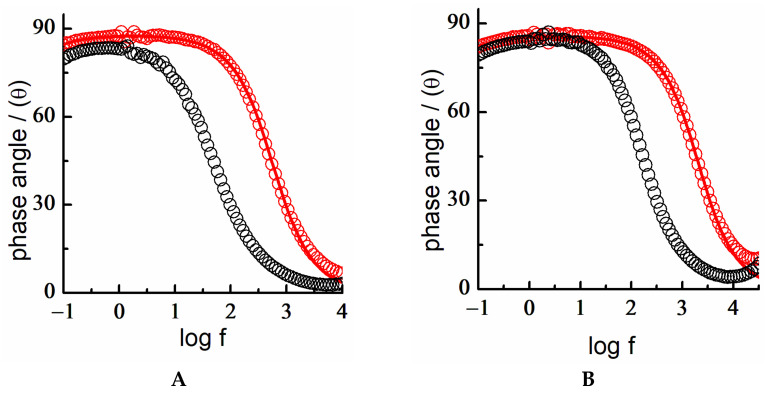
Electrochemical impedance spectra. Bode plot of Au 18K (black circle) and MUA-SAM on Au18K (red circle) in: (**A**) acidic media and (**B**) basic media. Fitting of experimental data to a [R(RQ)] equivalent circuit (red). E = −0.3 V.

The EIS study in both acidic and basic media indicated that an ordered layer of MUA with insulating properties was also successfully formed (Figure 12). However, when comparing this behavior with that of the DT-SAM, higher capacitance values, C_dl_ < 4.5 μF/cm^2^, and a phase angle below 85° at low frequencies were obtained for MUA-SAMs. This could be ascribed to the presence of ionizable end groups (-COOH) in the MUA monolayer [63,64]. The best fit of the Bode plot was obtained with an equivalent circuit that included a resistance element in parallel with the capacitance of the electrical double layer. This occurred in all the experimental conditions studied in the potential region of stability of the SAM, both in acidic and alkaline media. The structural organization of the alkyl chain, pending an ionizable end group (-COOH), and together with the possible effect of the applied DC potential suggested that ionic mobility took place on the SAM interface in such a way that ions either partially or totally penetrated through the SAM. This explained the increase in the interfacial polarity and permeability observed by the electrochemical data.

Therefore, the presence of the monolayers on the surface of the Au 18K alloy (Scheme 1) produced significant variation in the electrical properties of the interface in all cases, which was represented by significant changes in the equivalent circuit employed to model their behavior with respect to the naked surface. In general, as described, the system evolved toward a more homogeneous surface. Table 5 summarizes the circuits obtained from the fitting of the Bode plot curves.

The new equivalent circuit representing each SAM was also linked to greater protection of the alloy surface that would potentially lead to a decrease in bioactivity in the salt medium, according to the following relationship, eliminating the resistance of the solution, R_s_, for sake of simplicity.

C<Q<(QR)

**Figure 13:**
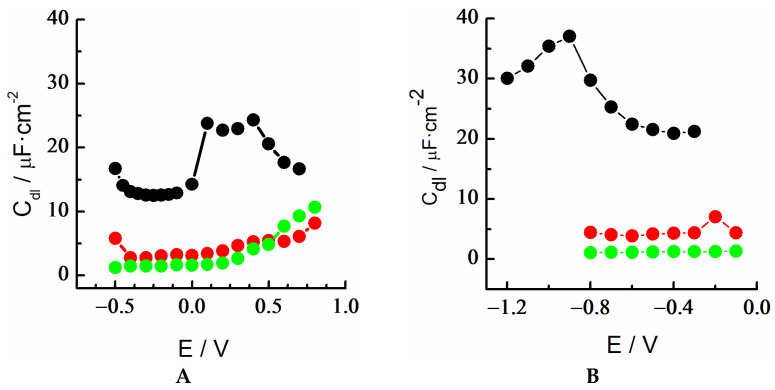
Variation of double-layer capacitance vs. potential of Au 18K (black); MUA-SAM/Au18K (red), and DT-SAM/Au18K (green) in (**A**) acidic medium and (**B**) basic medium.

Thus, the monolayers with 6MP and DT would be candidates to produce surfaces with low bioactivity, and therefore, stronger protection against corrosion processes. On the other hand, surface modification with MUA produced an important shield in the time scale less than 1 Hz and, even though ion mobility was still possible on the exposed surface of the SAM, the presence of the long alkyl chain (Scheme 1) suggested that may also act as a sufficiently protective barrier, depending on the experimental conditions. This was also based on the comparison of the evolution of the double-layer capacitance, C_dl_, obtained for both DT and MUA monolayers in the potential range studied (Figure 13). Overall, both SAMs showed an excellent blocking effect in a basic medium, without remarkable changes in the C_dl_ values within the potential interval of the monolayer’s stability.

On the contrary, in an acidic medium, there was a significant and gradual increase in double-layer capacitance at potentials greater than 0.4 V. This fact indicated that the application of potential induced defects in the SAMs, being particularly important for the DT-SAM at potentials greater than +0.8 V. Therefore, an anodic limit of stability for both types of SAMs on the alloy could be clearly established. These results could be a starting point for further studies to gain insight into the interpretation of the passivating effect of these SAMs in Au 18K against the corrosion observed with polarization curves and on its bioactivity in different jewelry pieces.

## 3. Materials and Methods

### 3.1. Chemicals

The 6-mercaptopurine (6MP), 1-decanethiol (DT), 11-mercaptoundecanoic acid (MUA), sodium hydroxide, semiconductor grade purity potassium hydroxide, and Triton X-100 were purchase from Sigma-Aldrich. Potassium chloride, perchloric acid, hydrochloric acid, and nitric acid were from Merck analytical grade. Acetone, sulfuric acid, potassium permanganate, and dihydrogen oxide water (33% w) were from Panreac. Ethanol and methanol were from Merck. All aqueous solutions were prepared with deionized ultrapure water produced by Millipore system.

### 3.2. Methods

A conventional three-electrode Metrohm electrochemical cell comprising a platinum coil as the counter electrode, a 50 mM KCl_(aq)_|Hg_2_Cl_2 (s)_|Hg_(l)_ (calomel electrode) as the reference electrode (E = +0.353 V vs. NHE), and a working electrode was used. As the working electrode, different gold and quaternary gold alloy samples were used and provided by the MAJ jewelry company. The gold sample was a homemade polyfaceted gold sphere, built by melting a gold wire (99.999%) to approximately reach a diameter of 2 mm, which was cleaned by flame-annealing and quenching in nitrogen degassed ultrapure water before use. The alloy samples were a selection of commercial gold quaternary alloys (Au-Ag-Cu-Zn), provided as flat square plates of 1 × 1 cm^2^ or 0.5 × 0.5 cm^2^, and wires with a length of 1 cm and 2 mm thick. The electrodes were electrochemically cleaned by potential cycling between −0.5 and 1.3 V in a 10 mM HClO_4_ solution until a steady-state and reproducible voltametric profile was obtained.

SAMs were formed by the immersion of clean substrates into aqueous, ethanolic, or micellar lyotropic solutions containing, unless otherwise stated, the specific adsorbate at a 1 mM concentration [64,69]. The adsorption time was varied at a controlled temperature, as indicated in the results and discussion sections.

Before the electrochemical experiments, the electrolyte solutions were kept under an inert atmosphere of nitrogen, where the gas was previously bubbled into the solutions for 15–20 min. Nevertheless, polarization curve studies were carried under atmospheric conditions. Cyclic voltammetry (CV) and electrochemical impedance spectroscopy (EIS) were performed on an Autolab (Ecochemie model Pgstat 20 or 30) instrument attached to a PC with the proper software (GPES, NOVA, and FRA) for total control of the experiments and data acquisition. EIS measurements were carried out at different contacted potentials (E_dc_) in a frequency range from 10 kHz to 0.1 Hz with a potential modulation amplitude (r.m.s.) of 5 mV.

The surface morphology and composition of the alloys were analyzed using scanning electron microscopy (SEM), energy dispersive X-ray spectroscopy (EDX), X-ray photoelectron spectroscopy (XPS) from SCAI, University of Cordoba, and X-ray fluorescence spectroscopy (XRF) from IQUEMA, University of Cordoba.

SEM micrographs and EDX microanalysis were obtained with a JEOL JSM7800F instrument equipped with a 138 eV RDX detector, and a 1.2 and 0.8 nm resolution at a 1 and 15 kV acceleration voltage, respectively.

Chemical composition spectra were acquired with a Rigaku ZSK Primus IV wavelength X-ray spectrometer. The system was equipped with a 3 kW Rh-target X-ray tube, ten analyzer crystals, a sealed proportional counter for the detection of light elements, and a scintillation counter for heavy elements.

X-ray photoelectron spectroscopy (XPS) analyses were performed with a SPECS Phoibos 150 MCD spectrometer using non-monochromatized (12 kV, 300 W) Mg KR radiation (1253.6 eV). Samples were mounted on a steel sample holder and directly introduced into the XPS analytical chamber. The working pressure was <5 × 10^−9^ Pa. The spectra were collected using a take-off angle of 45° with respect to the sample surface plane. The spectrometer was calibrated assuming the binding energy (BE) of the Au 4f_7/2_ line at 84.0 eV. The standard deviation for the BE values was 0.2 eV. Survey scans were run in the 0–1100 eV range (pass energy = 60 eV), while detailed scans were recorded for the Au 4f, Ag 3d, Cu 2p, and Zn 2p regions. The analysis involved Shirley background subtraction, and, whenever necessary, spectral deconvolution was carried out by nonlinear least-squares curve fitting, adopting a Gaussian sum function.

## 4. Conclusions

Quaternary gold alloys widely used in jewelry have been characterized by electrochemical and surface microanalysis techniques. It was verified that the heterogeneity and surface defects depended not only on the composition of the material, but also on the manufacturing treatment. For Au 18K plates, crystalline domains were formed with the practical absence of defects in the surface microstructure compared with the material fabricated as a wire. This influenced the corrosion behavior of the 18K alloys, whose resistance was significantly improved on a plate versus a wire piece.

The corrosion behavior of the alloys depended on their composition. The corrosion potential (E_corr_) increased as the proportion of Au does in the alloy. The electrochemical behavior of an 18 K gold plate alloy was similar to that of pure gold, mainly in an acidic medium. EIS spectra recorded in the double-layer region with the absence of faradic reactions, indicated that surface heterogeneity was almost completely masked in the presence of different types of thiol SAMs bearing aromatic rings (6MP-SAM) and long alkyl chains with methyl (DT-SAM) and carboxylic terminal (MUA) groups with different barrier thicknesses, ranging approximately from 6 to 16 Å.

SAMs were formed on 18K plate alloys, improving the insulating, blocking, and bioactive properties of the surface across a wide range of potentials where the layers were stable, and especially when the constituting SAM molecules bore chemically inert (non-ionizable) terminal groups in their structure. In the case of MUA SAMs, the effect of the ionization of the -COOH group and its influence on the intermolecular interactions manifested a non-ideal behavior of the interface that should be taken into account, depending on the working experimental conditions.

## Figures and Tables

**Table 1:** Composition of gold alloys (wt.%) by SEM-EDX.

Alloy/Element	Au	Ag	Cu	Zn
18K gold	77.1	11.7	9.3	1.9
14K gold	58.4	11.8	26.4	3.4
9K gold	41.0	8.2	44.1	6.7

**Table 2:** Composition of gold and gold alloys studied (wt.%) by XRF.

Alloy/Element	Au	Ag	Cu	Zn
24K	99.9	-	-	-
18K	74.3	11.8	12.1	1.7
14K	57.2	13.4	24.9	4.1
9K	36.6	13.6	42.4	7.4

**Table 3:** Composition of gold alloys studied (wt.%) by XPS.

Alloy/Element	Au	Ag	Cu	Zn
18K	69.22	20.82	9.32	0.65
9K	46.71	20.94	29.34	3.01

**Table 5:** Equivalent circuits of Au 18K and derivative SAMs.

Au 18K	Au 18K-6MP	Au 18K-DT	Au 18K-MUA
Rs (QR)	Rs C	Rs C	Rs Q → Rs (QR) ^1^

^1^ Change at f < 1 Hz in the Bode plot (see text).

## Data Availability

Not applicable.
